# Assessing Particle Release from Intraocular Lenses with a Combination of OptoFluidic Force Induction, μ-Raman and μ-FTIR

**DOI:** 10.3390/bioengineering12111138

**Published:** 2025-10-22

**Authors:** Andreas F. Borkenstein, Leon Ranz, Christian Neuper, Eva-Maria Borkenstein, Harald Fitzek

**Affiliations:** 1 Borkenstein and Borkenstein Private Practice, Privatklinik Der Kreuzschwestern Graz, Kreuzgasse 35, 8010 Graz, Austria; crustalith@gmx.at (A.F.B.);; 2 Institute of Electron Microscopy and Nanoanalysis (FELMI), Graz University of Technology, Steyrergasse 17, 8010 Graz, Austria; 3 Graz Centre for Electron Microscopy (ZFE), Steyrergasse 17, 8010 Graz, Austria; 4 Brave Analytics GmbH, Stiftingtalstraße 14, 8010 Graz, Austria

**Keywords:** microplastics, intraocular lenses, particle measurements, optofluidic force induction, vibrational spectroscopy

## Abstract

Intraocular lenses (IOLs) are among the most common medical implants that remain in the body long-term, with millions of IOLs implanted into patients every year. In addition, there are rapidly growing concerns about microplastic pollution, including particle emission from medical implants directly inside the body. Against this backdrop, we analyze the particle emission of seven common types of IOLs over a 30-day period under laboratory conditions. To accomplish both particle counting over a long period and chemical identification, we combine OptoFluidic Force Induction (OF2i), a novel online particle counting method, with micro Fourier Transform Infrared Spectroscopy with Attenuated Total Reflection (μ-FTIR-ATR) and Raman microscopy. Encouragingly, over the 30-day period, no significant particle emission from the IOLs was detectable. Neither was any increase in particle count detectable by OF2i, nor could any particle related to IOL material be found out of over 500 particles analyzed on non-control samples by FTIR and Raman microscopy. The most notable limitation of these results is the 30-day period, which is short compared to the time an IOL stays in the patient, which can be years or even decades. However, two of the tested IOLs were stored in liquid in their original packaging, the analysis of which represents a less-controlled long-term version of our study. Whilst microplastic contamination was found in these liquids, the FTIR and Raman analysis showed that it relates to the packaging materials (PE, PP) rather than the IOLs (acrylic), pointing to a high stability of the IOLs. Future work should try to assess longer time frames with accelerated aging (thermal/UV/oxidative conditions) to approximate long-term in vivo scenarios. Moreover, our findings highlight the need for manufacturers to ensure maximum stability of packaging materials and packaging methods to minimize potential microplastic contamination.

## 1. Introduction

Microplastics are ubiquitous in natural environments [1,2,3], and it has been estimated that up to 5 g are ingested by humans in a week [4]. Worryingly, various potential pathways for microplastics to cause harm, such as inflammatory response, initiation of signaling pathways responsible for malignant transformation, oxidative stress or metabolism disorders, have been described [5,6,7]. Recently, medical procedures and implants as potential sources of microplastic directly in the body have become a new research focus. A study on microplastics in the surgical environment has shown increased concentrations in the air during working hours [8]. Microplastic release from coronary catheters has been demonstrated under laboratory conditions [9]. A study on the microplastic release of sutures, again under laboratory conditions, unsurprisingly found a large number of particles released from absorbable sutures, but encouragingly no particle release from non-absorbable sutures over an 8-week period [10]. The release of microplastics, their prevalence and their hazardous effects are all the focus of currently ongoing research, and more studies will be necessary to provide a comprehensive picture. The aim of this study is to examine whether intraocular lenses (IOLs), one of the most commonly used implants in medicine, release particles.

IOLs are implanted over 20 million times every year worldwide [11,12]. They are used in ophthalmology primarily during cataract surgery, where the clouded natural lens is removed and an artificial lens (the IOL) is implanted. Another application is during refractive surgery, which can also involve replacing the clear, natural lens with an artificial lens to improve refraction and quality of vision [13]. IOLs are most commonly inserted into the capsular bag of the eye (posterior chamber lens) but can in principle be placed in different parts of the eye. An IOL consists of an optic, which is usually circular and has a diameter of 6.0 mm, and a haptic, which is used for fixation and stability in the eye. The total diameter of most posterior chamber lenses is between 12.5 and 13.0 mm. There is a variety of optical designs, from monofocal lenses with one focal point, and enhanced depth of focus lenses with improved depth of field, to trifocal and polyfocal optics, which are designed to provide sharp vision at different distances. In addition, there are toric lenses to correct astigmatism. Materials of intraocular lenses can be classified using their properties including Abbe number, biocompatibility, hydrophobicity and refractive index. This large variety of designs is accompanied by a large variety of materials used for IOLs, including collamer blends, PEG-PEA/HEMA/Styrene copolymer, polymethylmethacrylate (PMMA) and silicone [12,14]. Today, the most common implanted IOL material is acrylic, where there is a further distinction between hydrophobic and hydrophilic acrylic IOLs [15]. The new generation of foldable IOLs are composed of the methacrylate backbone of PMMA with additional hydroxyl groups introduced in the side chains [16,17]. The addition of hydroxyethylmethacrylate (HEMA), a material used in the manufacturing of contact lenses, poly (2-HEMA) or poly-HEMA confers flexibility to the IOL [17]. Flexibility is important for safe and easy implantation of the folded lens into the eye with the smallest possible incision sizes. This can reduce risks such as infection, endophthalmitis or the development of astigmatism or scars. Modern IOLs are usually preloaded in injectors to prevent further handling [18,19,20].

**Figure 1:**
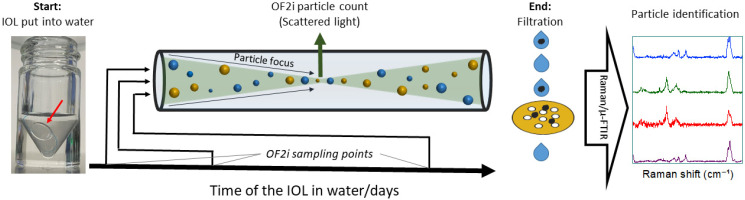
Combined OF2i, Raman microscopy and μ-FTIR approach to measure particle release from an IOL over a long period. Particle counts are made by OF2i, using only a small amount of liquid at specific sampling points during the experiment. Particle identification is performed by filtration and Raman microscopy, complemented by μ-FTIR, at the end of the experiment. Photograph courtesy of Christian Neuper. Copyright 2025.

In addition to the large variety of IOL designs and materials, there are two challenges when it comes to detecting particle release of IOLs. First, IOLs can remain in the body for years and even decades, so monitoring the particle release over long periods is necessary. Second, the amount of released particles over a period that is accessible under laboratory conditions is expected to be small. The combined quantification and identification of nanoplastics at low concentrations remains an ongoing challenge [21]. A recent extensive study of current detection methods concluded that any analytical technique on its own is limited, if a combination of morphological characterization, chemical identification and/or quantification is required, suggesting that increased use of the combination of several methods is the way forward [22]. In this work, we combine OptoFluidic Force Induction (OF2i), a novel online particle counting method, with μ-FTIR-ATR and Raman microscopy. As indicated in Figure 1, this combination allows us to monitor particle counts at different times during an experiment, whilst also identifying the particle composition at the end of the experiment.

OF2i is a particle counting method that is based on the combination of a microfluidic flow cell and a weakly focused vortex laser beam, which is used as a 2D optical trap. The vortex beam both guides particles to the detection spot and accelerates them relative to the flow speed in the microfluidic cell [23]. Based on the observation of single particles accelerating in the measurement spot, number concentrations and size distributions can be measured [24]. The trapping beam is generated by a linearly polarized 532 nm continuous-wave diode-pumped solid-state (CW-DPSS) laser with a maximum output power of 1.6 W (Laser Quantum, Stockport, UK). The beam is aligned using two high-precision mirrors and expanded by a factor of five to ensure optimal focusing conditions. A zero-order vortex half-wave plate (q = 1) converts the linearly polarized Gaussian beam into an azimuthally polarized Laguerre–Gaussian mode with a topological charge of m = 2. This beam is then focused into the center of the flow channel, forming a 2D optical vortex trap within the measurement cell. A microfluidic pump connected to the cell provides a flow rate of 4 μL/min. The light scattered by the trapped particles is then collected, magnified and recorded using a custom-built ultramicroscope imaging system. OF2i was developed as an online tool for nanoparticle characterization and, most importantly for this work, only uses small amounts of liquid during the measurement [25]. Furthermore, successful hyphenation to both Raman spectroscopy and ICP-MS has been demonstrated [26].

Both μ-FTIR-ATR and Raman microscopy are well established tools for the identification of micro- and to some extent nanoplastics [27]. Their main advantage is that plastic particles, as well as non-plastic contamination particles, can be unambiguously identified without the use of any labeling [28]. Their main disadvantages are that sample preparation, most typically filtration for particles in liquid, is required, making continuous measurements impossible, and that the measurement of large numbers of particles is rather time consuming [29,30]. A combination of both methods is recommended, in order to achieve the highest accuracy for unknown particles [31]. In this work we use Raman microscopy as the main spectroscopic method, since it offers a higher spatial resolution, allowing for the measurement of particles down to approximately 500 nm. As high-fluorescent can mask the Raman spectrum for some particles, μ-FTIR-ATR is used as a complementary method to improve the accuracy of the particle identification.

## 2. Materials and Methods

### 2.1. Intraocular Lenses

In our experiment we have tested the following intraocular lenses: A: Alcon AcrySof (Alcon, Fort Worth, TX, USA), B: Bausch & Lomb enVista (Bausch & Lomb, Bridgewater, CT, USA), C: Hoya Vivinex iSert (Hoya, Tokyo, Japan), D: Johnson & Johnson Tecnis 1 (Johnson & Johnson, New Brunswick, NJ, USA), E: Rayner RayOne (Rayner, Worthing, UK), F: Zeiss Meditec CT Lucia 621P (Zeiss, Jena, Germany) and G: Ophtec Artisan (Ophtec, Groningen, The Netherlands).

The IOLS A–D and F are hydrophobic acrylic IOLs; lens E is a hydrophilic acrylic IOL. These samples are foldable, one-piece IOLs for the posterior chamber. Sample G is an anterior chamber lens, made of PMMA CQ-UV. The most obvious difference in these acrylic lenses at a chemical level is the water content, with hydrophilic usually comprising 5–38% water content, compared to ≤5% for hydrophobic. Another difference in IOL models is the manufacturing process (molded versus lathe-cut). However, most details on material properties are kept secret by the companies. The posterior chamber lenses A, C, D and F have an optical diameter of 6.0 mm and an overall diameter of 13.0 mm. Samples B and E have an optical diameter of 6.0 mm and an overall diameter of 12.5 mm. Sample G as an anterior chamber lens has an optical diameter of 5.4 mm and an overall diameter of 8.5 mm. The tested lenses have a similar refractive index of 1.46–1.49 and an Abbe number of 51–56.

### 2.2. Sample Preparation for the OF2i Measurements

For the long-term OF2i measurements, the samples were stored in water in 20 mL glass vials(Chromatographie Handel Müller, Fridolfing, Germany). The water for the long-term OF2i measurements was specially prepared, to ensure as low a native particle number as possible. To this end, water from an ANDRONA AQUA-Lab INTEGRITY+ (Aqua-Lab, Westerburg, Germany) water purification system was additionally filtered through a Whatman Anotop 25 0.02 μm filter (Whatman, Maidstone, UK) using a BRAUN Injekt Luer Solo 5 mL syringe (Braun GmbH, Kronberg, Germany). Note that a fresh syringe/filter was used for each sample. Water prepared this way was also used to flush the OF2i instruments between measurements. Ultrapure, 0.02 μm-filtered water was used to minimize background counts and to decouple packaging-derived particulates from any potential IOL matrix release under strictly controlled conditions. This isolates instrument and packaging contributions but does not reproduce the biomolecular milieu of the anterior chamber, where proteins and lipid-associated species are present and can adsorb to polymer interfaces.

The vials were prepared with a cleaning protocol that ensured a low particle count from contamination. To start with, vials were rinsed using water from an ANDRONA AQUA-Lab INTEGRITY+ water purification system. After that, they were completely filled with the same purified water before being placed into an Elma TRANSSONIC T 460 ultrasonic bath (Elma Schmidbauer GmbH, Singen, Germany) for 15 min, in order to loosen contaminants adhered to the walls of the vials. The vials were then rinsed again with purified water. Every rinsing step was repeated four times. Next, the vials were rinsed using both purified and filtered water, produced as described above. Finally, the vials were filled with 4 mL of specially prepared water, as described above. This was the final preparation step for the control samples. Note that every IOL sample was paired with one empty control sample. As for the vials meant to receive the IOLs, they were measured using OF2i to confirm a low particle count, and before measurement they were placed in the ultrasonic bath for 2 min. At the start of the OF2i measurements (first measurement), the IOLs were removed from their packaging using clean metal tweezers, dipped into 20 nm filtered water in order to rinse off possible surface contaminations and then placed into the water. In between the OF2i measurements, all samples were kept refrigerated in order to inhibit microbial growth in the water.

Three positive control samples, spiked with polystyrene beads of a known concentration and size, were prepared as well. To achieve this, three polystyrene samples with nominal particle sizes of 600 nm, 2000 nm and 5000 nm were diluted in high-purity MilliQ water (Merck KGaA, Darmstadt, Germany), which was additionally filtered through a 20 nm Whatman Anotop 25 (0.02 μm) filter using a BRAUN Injekt Luer Solo 5 mL syringe. The 600 nm (±9 nm) polystyrene nanospheres (Nanosphere Standards, NIST-traceable, Polymer (Polystyrene) Microspheres in Water, 15 mL, 1% solids (*w*/*w*)), purchased from Thermo Scientific (Thermo Scientific, Waltham, USA), were diluted by a factor of 1:8 × 10^6^ to achieve a final concentration of approximately 10,500 particles per milliliter. However, the 2000 nm (±38 nm) and 5000 nm (±140 nm) diameter Polystyrene microspheres (Applied Microspheres 2000 Series), NIST-traceable particle size and count control, polymer particle suspension, number of particles: 10^7^/mL (±10%)) were diluted by a factor of 10^3^ to obtain a final concentration of 10,000 particles per milliliter.

### 2.3. OF2i Measurements

OF2i measurements were performed using a BRAVE B-Curious system from BRAVE Analytics. Raw video data was recorded at 200 frames per second and processed using the BRAVE Analytics software suite, Hans 2.4. All measurements were conducted with the Nanoset 3 configuration, which is optimized for detecting and counting particles down to 100 nm. The key parameters for this setup were a laser power of 1500 mW (at 532 nm), a flow rate of 4 μL/min, a total measurement time of 1 h, a camera exposure time of 3000 μs and a gain of 10. For the spiked samples, a minimum particle size threshold of 400 nm was applied during particle count calculations to exclude potential contamination nanoparticles of inappropriate sizes. For the IOL and reference sample measurements, a threshold of 100 nm was used, as smaller particles do not exhibit sufficient acceleration to allow for accurate size and count determinations.

### 2.4. Sample Preparation for Microscopic Measurements

For the microscopic measurements, the IOLs were removed from the water using a clean metal tweezer and the samples (water) were filtered onto Au-coated PC filters using a Millipore vacuum filtration setup (Merck KGaA, Darmstadt, Germany). Millipore ISOPORE 0.05 μm filters, coated with 100 nm gold using a Leica EM ACE600 sputter coater (Leica Microsystems, Wetzlar, Germany), were used for the IOLs Bausch & Lomb enVista, Zeiss Meditec CT Lucia 621P, Hoya Vivinex iSert, packaging liquid from the Bausch & Lomb enVista and control samples 1–3. As Millipore ISOPORE 0.05 μm filters were not commercially available at the time, i3 TrackPor 0.1 μm filters (Au-coated PC filters, i3 Membrane GmbH, Radeberg, Germany) were used for the IOLs Johnson & Johnson Tecnis 1, Ophtec Artisan, Alcon AcrySof, Rayner RayOne, packaging liquid from Rayner RayOne, control samples 4–7 and all spiked samples. Right before the filtration, the samples were placed in an ultrasonic bath for 2 min. The filter was placed on the mesh of the vacuum filtration setup, and the funnel was rinsed with purified water and ethanol before being dried with pressurized CO_2_ and placed on the filter. A standard 1” SEM stub was covered with conductive tape, and an aluminum disk with a diameter of 1 cm and a thickness of 3 mm was placed in the center of the stub on top of the tape. After all the liquid had passed through, the filter was mounted to the stub in a way that created a flat surface over the disk. This flat surface is highly desirable for light microscopy.

### 2.5. Raman Microscopy

Raman microscopy of the particles was performed using a Horiba LabRAM HR 800/Olympus BX 41 microscope(Horiba, Kyoto, Japan) with a 532 nm laser. The filter was scanned manually for particles using the light microscope and Raman spectra of individual particles were measured manually. An automated measurement was not efficiently possible due a combination of a sparse distribution of particles, a non-uniform appearance in the light microscope (making automated particle recognition challenging) and low Raman scattering from a portion of particles. Since different objective lenses are optimal for different particle sizes, the particle distribution were roughly split into particles larger than 10 μm and particles smaller than 10 μm. An Olympus MPlanN 10× objective (NA 0.25) (Olympus, Tokyo, Japan) was used for the large particles and an MPlanN 100× objective (NA 0.9) was used for the small particles. The laser power and integration time was adjusted on a particle-by-particle basis to ensure sufficient spectral quality whilst not damaging particles. In each size category, at least 20 particles were measured per sample for a total of at least 40 particles per sample. All data treatment, such as baseline corrections, was performed using the LabSpec 6 software (Horiba, Kyoto, Japan), and spectral interpretation was performed based on an internal database and the commercial KnowItAll software/database (Wiley Science Solutions, Hoboken, NJ, USA).

### 2.6. FTIR Microscopy

Fourier Transform Infrared (FTIR) microscopy was performed using a Bruker Hyperion 3000/Tensor 27 microscope (Bruker, Billercia, MA, USA), with a 20× ATR (Ge crystal) objective(Bruker, Billercia, MA, USA). The filter was scanned manually for particles using the light microscope and FTIR-ATR measurements of individual particles were measured manually. The scan number was 32 and the reference measurement was performed with the ATR crystal in air. Since the samples need to be in contact with the crystal, causing potential damage to the filter and a loss of the particle, the FTIR measurements were performed last on each sample. Similarly, for the Raman measurements, the particles larger than 25 μm were measured differently from particles smaller than 25 μm, for practical reasons. For the large particles, a single element MCT detector(Bruker, Billercia, MA, USA) and an adjustable aperture was used; this setup produces a better signal (for large particles) and offers a broader spectral range (4000–600 cm^−1^) compared to using the FPA detector. The small particles were measured by acquiring a spectral image using a 64 × 64 element FPA detector(Bruker, Billercia, MA, USA) and extracting the spectra from this image. This approach has a far superior spatial resolution compared to the MCT/aperture approach but only offers a limited spectral range (4000–900 cm^−1^). For each sample at least 5 “small” and 10 “large” particles were measured for a minimum of 15 particles per sample. All data treatment, such as baseline corrections, was performed using the OPUS software ((Bruker, Billercia, MA, USA)) and spectral interpretation was performed based on an internal database and the commercial KnowItAll software/database (Wiley Science Solutions, Hoboken, NJ, USA).

## 3. Results

### 3.1. OF2i Particle Counts

The results of the OF2i measurements immediately after the IOLs were put into the highly pure water and 30 days later are summarized in Table 1. No significant difference was found between the IOL samples and control samples, both at the start of the measurements and after 30 days. For both the IOL and control samples the average particle count is close to zero (≈0.3) at the start, increasing slightly to about one (≈1.4) after 30 days. The highest count for any IOL sample (Rayner RayOne) is two at the start and six after 30 days, not much different from the highest count for a reference sample (1 at the start and four after 30 days). The storage liquid of the Bausch & Lomb enVista is also in the same range, with three particles. The only sample with a significantly larger particle count is the storage liquid of the Rayner RayOne at 21 particles, which, as shown by Raman and FTIR microscopy, is due to particles from the IOL packaging, not the IOL itself.

### 3.2. Raman and FTIR Microscopy

**Figure 2:**
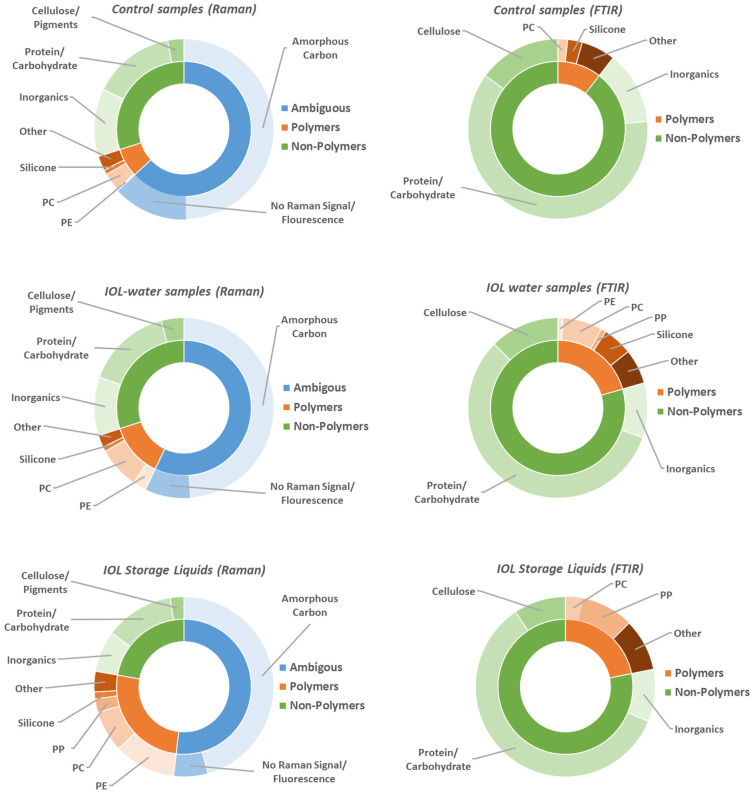
Summary of the spectroscopic analysis of the particles after 30 days. (**Left**) Raman microscopic analysis with a focus on small particles (<10 μm): (**top**) control samples (total: 306 particles), (**middle**) IOL water samples (total: 313 particles), (**bottom**) IOL storage liquids (total: 81 particles). (**Right**) FTIR-ATR microscopic analysis with a focus on large particles (>10 μm): (**top**) control samples (total: 113 particles), (**middle**) IOL water samples (total: 116 particles), (**bottom**) IOL storage liquids (total: 31 particles). The full dataset, broken down to individual samples, can be found in Table S1 (Raman) and Table S2 (FTIR).

With the exception of the Rayner RayOne Storage Liquid, no increased particle counts were found in the OF2i measurements. The main questions in the Raman and FTIR measurements are, therefore, can we find any particles from the IOLs at all, and what is responsible for the higher particle count in the storage liquid? Since automated measurements were not possible, as described in the method section, manually at least 40 particles (mostly <10 μm) were measured with Raman microscopy and 15 particles (mostly >10 μm) with FTIR microscopy for each sample. Note that the particles were sparsely distributed and that the filters appeared almost empty under the light microscope. The results of the Raman and FTIR measurements are summarized in Figure 2 by sample type (control samples, IOL water sample and IOL storage liquid). The full dataset can be found in Table S1 (Raman) and Table S2 (FTIR) in the Supplementary Material.

Out of a total of more than 500 particles analyzed by FTIR and Raman microscopy on non-control samples, not a single particle of the base material of any IOL was found. These results are consistent with the OF2i results, where no increased particle count was found of IOL water samples compared to the control sample. Interestingly, for the IOL storage liquids, which represent a sort of long-term version of our experiment for the two IOLs that are stored in liquid (Bausch & Lomb enVista and Rayner RayOne), we found an increased proportion of PE and PP particles. These particles are likely connected to the packaging used for the IOL, as the general packaging and holder the Bausch & Lomb enVista and Rayner RayOne are sold in contain PE and PP. Note that packaging particles were found for both storage liquids, whereas only the Rayner RayOne Storage Liquid had a significantly higher particle count in the OF2i. The presence of plastic particles from the packaging in the storage liquid, but not particles from the IOLs themselves, might be due to the much larger surfaces area of the packaging, which is especially true for the Rayner RayOne, where the storage liquid covers the entire injections system. However, it could also indicate a higher stability of the IOL material.

In terms of particles found by Raman microscopy, we have categorized the results into polymer particles, non-polymer particles and ambiguous particles. Non-polymer particles are a mixture of contaminations that can found both in the liquid or on the empty filters. The main categories are cellulose/pigments, biological materials (protein, carbohydrates) and inorganics. Note that cellulose lamina are used as “separation disks” in the packaging of the PC filters. Biological materials could result from limited bacterial growth during the storage period or contamination of the filter, and the inorganics found are typical dust particles.

Polymer particles are the main category we are interested in, as this is where particles from the IOLs would show up. Two notable polymers that are found are polycarbonate (PC) and silicone. PC is the material the filters are made of and indicates a rip in the gold coating or perhaps some contamination from the filter storage, as the filters are stacked in their packaging with cellulose lamina in between. Silicone is used for the syringe penetrable caps of the glass vials. Furthermore, PE and PP are found in the packaging of the IOLs and in a higher concentration in the storage liquids. The polymers categorized as other contain singular contaminations with other polymers such as PA, PET, PU, etc., of unknown origin, but typically only one or two particles of each type were found across all samples.

Ambiguous particles are particles that have either no Raman signal, a too high fluorescent background for Raman measurement or amorphous carbon particles. Whilst no identification of particles without Raman signal or prohibitively high fluorescents is possible, these are unlikely to originate from the IOLs, as the base material of all IOLs has a clearly distinct Raman signal with limited or no fluorescents. The amorphous carbon particle representing almost half of the particles found by Raman are more difficult to interpret. On one hand, it is a very common material to be found, for instance in organic matter or common dirt, and Raman spectroscopy is highly sensitive to amorphous carbon, so it could be simply a contamination, an interpretation that is also bolstered by the high content in the control samples. On the other hand, it can also result from laser damage to the sample, and although the laser power was kept as low as possible, it cannot be excluded that some amorphous carbon particles are the result of laser damage, potentially masking a population of IOL particles that happens to be particularly beam sensitive.

FTIR microscopy does not suffer from fluorescent background or beam damage, as such it can serve as a further probe into the ambiguous category. Furthermore, Raman spectroscopy and FTIR spectroscopy have different levels of sensitivity for different bonds and therefore different materials. Using both helps to avoid selection bias based on the method specific sensitivity. Two things are especially important to consider for the comparison between the FTIR- and Raman-results. First, Raman microscopy has a better spatial resolution and the Raman measurements were therefore focused on small particles (<10 μm), whereas most particles measured with FTIR are larger than 10 μm. Second, FTIR is insensitive to amorphous carbon. Apart from the elimination of the ambiguous category, the FTIR results have been categorized in the same manner as the Raman results, into polymer and non-polymer, with the same respective subcategories. Overall, it has to be emphasized that the FTIR results confirm the main conclusions of the Raman results. Firstly, no particles from the IOLs themselves were found in any measurement. Second, in the IOL storage liquids there is a higher proportion of polymeric particles attributable to the packaging of the IOLs. Third, apart from the different proportions found between non-polymer to polymer particles, which is due to the elimination of the ambiguous category and the different size regime probed, the contamination particles are attributable to the same sources as in the Raman measurements.

Finally, in the analysis of the spiked samples, using Raman microscopy the PS particles of all sizes (0.6 μm, 2 μm, 5 μm) could easily be found and identified, whereas using FTIR microscopy, only PS particles down to 5 μm could be readily detected and identified. PS particles with a diameter of 2 μm were still detectable by FTIR, but only with great difficulty. We assess that without prior knowledge of what to look for, particles of this size would probably be missed by FTIR microscopy in our experiment, and therefore view a diameter of 5 μm as the lower size bound for the FTIR measurements. Figure S1 (Supplementary Material) shows examples of the spiked particles detected by Raman microscopy and FTIR microscopy.

## 4. Discussion and Limitations

The primary aim of this study was to evaluate the particle emission from intraocular lenses (IOLs). To this end, a novel approach combining OF2i, an online particle monitoring method, with post-mortem Raman and FTIR microscopy for particle identification was implemented. Assessing the performance of this new combination is the secondary aim of this study.

Seven different types of common IOLs were tested over a 30-day period and no particles originating from the IOLs themselves could be found. It has to be stated that the 30-day study period is relatively short compared to the lifespan of IOLs in patients, which can be several years or decades. However, extending the study duration would pose significant challenges. Apart from logistical challenges, the most notable of these challenges is microbial growth that cannot be avoided without adding stabilizing agents that in themselves might interfere with the particle measurements. Even with careful sample preparation and refrigeration, microbial contamination issues were already observed in this short-term study. This study intentionally focused on a 30-day observation window to demonstrate proof-of-concept performance and early post-handling stability; however, the duration is short relative to the decades-long in vivo lifetime of IOLs, which we explicitly acknowledge as a limitation. Accordingly, the findings support short-term stability but do not address the multi-year in vivo lifetime of an IOL. Therefore, slow degradation pathways (e.g., hydrolysis, UV/photo-oxidation, mechanical/micro-wear aspects) that may manifest over longer timescales cannot be excluded.

Over the multi-year lifetime of an IOL in vivo, polymer degradation may arise from several concurrent mechanisms. Acrylic and silicone matrices are generally resistant to hydrolysis, yet slow ester-cleavage reactions can occur under continuous exposure to the oxidative, protein-rich aqueous humor. In addition, photo-oxidative aging driven by UV and high-energy blue light, as well as thermo-mechanical stress from temperature and intraocular pressure fluctuations, may alter polymer cross-linking and/or generate nanoscale debris. Enzymatic activity (e.g., esterases, lipases) and reactive oxygen species originating from ocular metabolism could further accelerate surface oxidation or chain scission. Quantitative assessment of these processes will require extended soak tests combined with accelerated-aging protocols—elevated temperature, UV irradiation and oxidative media—complemented by periodic OF2i monitoring and post-mortem μ-Raman/μ-FTIR analysis. Such experiments could further enable estimation of long-term release kinetics and mechanistic differentiation between physical abrasion and chemical degradation, aligned with the risk-based expectations for long-term implantable devices under the EU MDR.

However, two IOL types (Bausch & Lomb enVista and Rayner RayOne) that are stored in stabilized water in their original packaging give us a chance to examine a longer time-frame, as IOLs can be stored in their packaging for several months before use. Of course, this “storage” water is a less-controlled experimental condition. Notably, one of the “storage” waters (Rayner RayOne) had a higher particle count when compared to the blank samples, but the Raman and FTIR analysis showed that the plastic particles in it originate from the packaging material (PE, PP) rather than the IOLs themselves (acrylic). Notably, particles from the packaging material (PE, PP) were also found in the other “storage” water (Bausch & Lomb enVista) by Raman and FTIR analysis, despite no higher particle count in the OF2i. This is possibly attributable to the larger surface area covered by the “storage” water for the Rayner RayOne, where the entire injection system is immersed in the water. This again points to a high stability of the IOLs.

Another limitation of this study is that the IOLs were stored in undisturbed pure water for the duration of the experiment. Therefore, there are no degradation mechanisms that IOLs would experience in the human eye, such as UV exposure, mechanical stress or biological interactions [32]. Further studies would be useful incorporating these variables to provide a more comprehensive assessment of IOL durability. While the panel of IOLs (*n* = 7 models spanning hydrophobic/hydrophilic acrylic and PMMA) cannot capture all market variants, the multi-modal results were consistent across models. However, larger-scale studies are warranted to confirm generalization.

In terms of assessing the detection method, the blank samples consistently showed particle counts close to zero in the OF2i. In addition, the particles identified by Raman and FTIR analysis on the blanks are attributable to the vials used for storage, the filtration equipment or dust particles. Note that no cleanroom facilities were available for the sample preparation or filtration. The positive samples, spiked with 0.6 μm, 2 μm and 5 μm PS-particles, show a detectable particle count above the blank samples at concentrations as low as 10^4^ particles/mL in the OF2i measurement. The spiked PS particles were readily detectable by FTIR and Raman microscopy down to 5 μm and 0.6 μm, respectively.

Compared to relying solely on ex situ filtration followed by μ-Raman or μ-FTIR analysis, which provides chemical identity but lacks temporal resolution and is insensitive to transient or sub-micron particle dynamics, the integration of OF2i monitoring enables additional continuous, label-free tracking of single particles in liquid. It also improves both concentration and size limitations for detection. The combined workflow thus bridges quantitative particle kinetics with chemical specificity, establishing a transferable framework for future implant-material studies where both count evolution and molecular composition are critical.

## 5. Conclusions and Outlook

Modern intraocular lenses appear to be very stable with respect to particle emission in the short term, especially compared to other materials. There was no difference found in the particle emission of hydrophobic and hydrophilic IOLs. In the IOL storage liquids, only packaging material but no IOL particles were found.

The detection of polyethylene and polypropylene fragments in storage liquids emphasizes that the packaging environment itself can contribute measurable microplastic contamination. Such particles likely arise from abrasion or stress cracking of polymer components during sterilization, transport or long-term storage. While they are chemically inert and distinct from the IOL matrix, any particle transfer to the surgical field could be minimized through improved polymer formulation, surface finishing or pre-implant rinsing protocols. Further studies should systematically assess these possible effects, and, in close collaboration with manufacturing companies, develop optimized packaging concepts to minimize such risks in the future.

Future research should also address the long-term behavior and effect of degradation mechanisms to further refine our understanding of IOL stability and potential particle emissions. Further investigations could be carried out with regard to the packaging techniques of IOLs and the specific storage liquids used, to find out whether rinsing these IOLs with sterile balanced salt solution (BSS) before the actual implantation process would be recommended to avoid or reduce particle contamination in the eye.

Moreover, as a possibility for a long-term study in the future, it could be considered to examine aqueous humor fluid for nano/microplastics in eyes that have had IOLs implanted for many years. This would be particularly interesting in order to carry out specific and well-designed studies to determine whether there are connections to pathophysiological processes, such as inflammatory reactions, wound healing disorders, changes in intraocular eye pressure, development of postoperative edema, secondary cataract or any clouding/opacities of the IOL itself. However, this also raises more questions of what other factors need to be taken into account, as humans have other potential micro/nanoplastics uptake possibilities via the gastrointestinal tract, the skin or other routes that have been described.

A clinically relevant damage source in this context that should be studied are Nd:YAG laser shots/pits in the material. These can occur during Nd:YAG laser capsulotomy, which is used to treat posterior capsule opacification, a common and typical consequence after cataract surgery. It has been shown that an incorrectly aimed laser beam can damage the IOLs [33,34,35], possibly resulting in fragments that are then released into the eye in a concentrated burst. The authors are already conducting studies on this important topic and results should be available soon.

Next steps regarding the evaluation of microplastic release over time should include artificial-aqueous-humor and protein-supplemented balanced-salt-solution studies [36] under controlled light/temperature/flow conditions, followed by in vivo validation in rabbit eyes [37], to more realistically bracket long-term IOL stability while maintaining chemical identification by techniques like μ-Raman/μ-FTIR-ATR and online particle monitoring by OF2i.

## Figures and Tables

**Table 1:** OF2i particle counts at the start of the experiment and after 30 days. Measurements were performed in two 30-day measurement batches.

Sample	Start/#P	30 Days/#P	
Batch 1			
Bausch & Lomb enVista	0	0	
Bausch & Lomb enVista Storage Liquid	3	-	
Zeiss Meditec CT Lucia 621P	0	1	
Hoya Vivinex iSert	0	0	
Control sample 1	0	0	
Control sample 2	0	1	
Control sample 3	1	2	
Batch 2			
Johnson & Johnson Tecnis 1	0	3	
Ophtec Artisan	0	0	
Alcon AcrySof	0	0	
Rayner RayOne	2	6	
Rayner RayOne Storage Liquid	21	-	
Control sample 1	1	4	
Control sample 2	0	0	
Control sample 3	0	0	
Control sample 4	0	3	
Spiked samples			
Polystyrene Ø 0.6 μm (1.05 × 10^4^ particles/mL)	21	-	
Polystyrene Ø 2 μm (10^4^ particles/mL)	23	-	
Polystyrene Ø 5 μm (10^4^ particles/mL)	19	-	

Note that the low count statistic, due to the highly pure sample preparation and seemingly negligible particle emission by the IOLs, are atypical for OF2i, and were usually statistics measured from at least several 100 particles. We therefore cannot give precise concentration numbers, as effectively no particles were found. As an order of magnitude estimate, based on the amount of liquid measured and the capture probability of the OF2i, we would expect 10^3^ particles/mL to result in roughly two particles counted. This is calculation is confirmed by the spiked samples, where around 20 particles were found for a concentration of 10^4^ particles/mL. The particle concentration for all IOLs and control samples is therefore around 10^3^ particles/mL or lower.

## Data Availability

The raw data supporting the conclusions of this article will be made available by the authors on request.

## Supplementary Materials

The following supporting information can be downloaded: https://www.mdpi.com/article/10.3390/bioengineering12111138/s1. Figure S1: Raman- and FTIR-microscopy of spiked samples (positive control): (a) Light microscopy of PS-beads (left to right 5 μm, 2 μm 0.6 μm); (b) Raman spectra of PS-beads; (c) Light microscopy of PS-beads (FTIR-ATR-objective); (d) FTIR-spectra of PS-beads (top: 2 μm, bottom: 5 μm).; Table S1: Summary of the Raman microscopy results for each individual sample.; Table S2: Summary of the FTIR-microscopy results for each individual sample.
