# Supplementary material for: Assessing Particle Release from Intraocular Lenses with a Combination of OptoFluidic Force Induction, μ-Raman and μ-FTIR
Source: Bioengineering (Basel). 2025 Oct 22;12(11):1138. doi: 10.3390/bioengineering12111138 (PMC12649333; doi:10.3390/bioengineering12111138)
Supplement: Supplementary file 1 [file bioengineering-12-01138-s001.zip › bioengineering-3914133-supplementary.pdf]

Assessing particle release from intraocular lenses with a combination of OptoFluidic Force Induction, µ-Raman and µ-FTIR (Supporting Information)

Andreas F. Borkenstein[1,•], Leon Ranz[2,•], Christian Neuper[3,4], Eva-Maria Borkenstein[1] and Harald Fitzek[2,3,*]

[1]Borkenstein and Borkenstein Private Practice, Privatklinik Der Kreuzschwestern Graz, Kreuzgasse 35, 8010, Graz, Austria

[2]Institute of Electron Microscopy and Nanoanalysis (FELMI), Graz University of Technology, Steyrergasse 17, 8010, Graz, Austria

[3]Graz Centre for Electron Microscopy (ZFE), Steyrergasse 17, 8010, Graz, Austria

[4]Brave Analytics GmbH, Stiftingtalstraße 14, 8010 Graz, Austria

[•]Equality contributed

[*]Correspondence author; Email: harald.fitzek@felmi-zfe.at

Number of tables: 2 (page 2-3)

Number of figures: 1 (page 4)

Summary of the Raman microscopy results

Table S1: Summary of the Raman microscopy results for each individual sample.

Sample	Polymers						Ambiguous			Non-Polymers			
	PE	PC	PP	Silicone	Other	total	Amorphous Carbon	No Raman Signal/ Flourescence	total	Inorganics	Protein/ Carbohydrates	Cellulose	total
Batch 1													
Bausch + Lomb enVista	7	3	0	0	0	10	22	10	32	5	14	3	22
Bausch + Lomb enVista Storage Liquid	9	1	0	0	2	12	19	3	22	4	5	1	10
Zeiss CT Lucia	0	0	0	0	2	2	32	6	38	8	8	4	20
Hoya Vivinex iSert	0	0	0	0	3	3	26	3	29	4	9	6	19
Control sample 1	0	0	0	1	0	1	22	11	33	8	12	4	24
Control sample 2	0	4	0	0	2	6	25	8	33	1	8	0	9
Control sample 3	0	1	0	0	3	4	18	10	28	10	6	4	20
Batch 2													
Johnson & Johnson Tecnis 1	0	9	0	0	0	9	26	2	28	9	5	1	15
Ophtec Artisan Aphakia	1	5	0	0	3	9	16	2	18	5	12	1	18
Alcon AcrySof Aspheric	1	6	0	1	0	8	28	4	32	4	5	0	9
Rayner RayOne Aspheric	0	5	0	2	0	7	31	3	34	4	4	0	8
Rayner RayOne Aspheric Storage Liquid	0	5	2	1	1	9	18	2	20	2	5	1	8
Control sample 1	0	0	0	0	0	0	28	4	32	5	3	1	9
Control sample 2	0	3	0	1	2	6	25	3	28	3	11	1	15
Control sample 3	1	1	0	1	0	3	22	6	28	9	7	0	16
Control sample 4	0	2	0	0	2	4	31	4	35	6	4	0	10

Summary of the FTIR-microscopy results

Table S2: Summary of the FTIR-microscopy results for each individual sample.

Sample	Polymers						No Signal	Non-Polymers			
	PE	PC	PP	Silicone	Other	total		Inorganics	Protein/ Carbohydrates	Cellulose	total
Batch 1											
Bausch + Lomb enVista	0	0	0	0	1	1	0	1	13	3	17
Bausch + Lomb enVista Storage Liquid	0	0	0	0	2	2	0	3	10	2	15
Zeiss CT Lucia	0	2	0	0	1	3	7	0	11	1	12
Hoya Vivinex iSert	0	1	0	0	3	4	0	2	10	3	15
Control sample 1	0	1	0	0	0	1	0	2	12	3	17
Control sample 2	0	0	0	0	1	1	0	4	7	5	16
Control sample 3	0	1	0	0	0	1	6	3	7	6	16
Batch 2											
Johnson & Johnson Tecnis 1	0	2	0	0	0	2	0	1	11	1	13
Ophtec Artisan Aphakia	0	1	1	0	1	3	0	1	9	3	13
Alcon AcrySof Aspheric	1	1	0	3	1	6	0	1	5	3	9
Rayner RayOne Aspheric	0	1	0	3	0	4	2	5	5	0	10
Rayner RayOne Aspheric Storage Liquid	0	1	3	0	1	5	0	0	9	1	10
Control sample 1	0	0	0	0	3	3	0	0	11	2	13
Control sample 2	0	0	0	0	3	3	0	3	8	1	12
Control sample 3	0	0	0	2	0	2	0	2	12	0	14
Control sample 4	0	0	0	1	0	1	0	1	13	0	14

FELMI-ZFE

Example FTIR/Raman-microscopy PS-beads

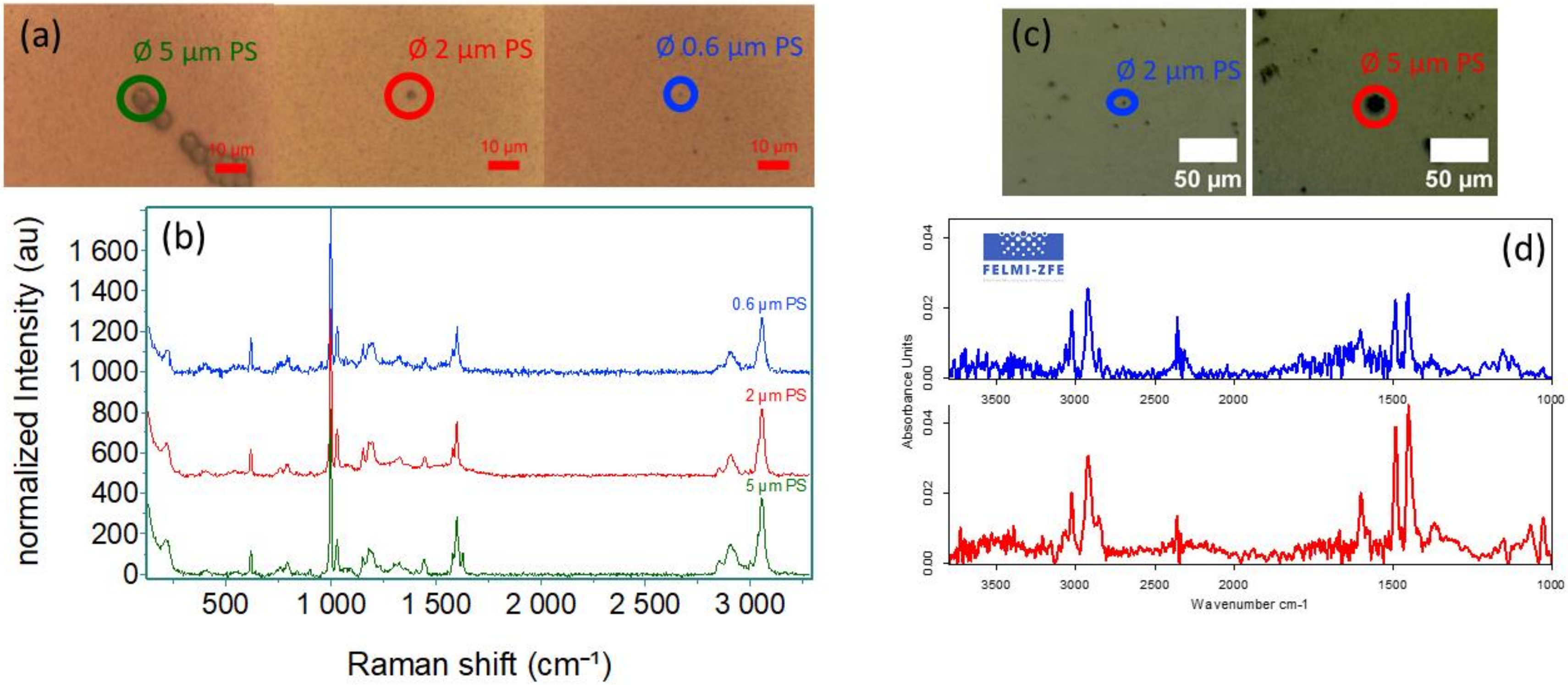

Figure S1: Raman- and FTIR-microscopy of spiked samples (positive control): (a) Light microscopy of PS-beads (left to right 5 µm, 2 µm 0.6 µm); (b) Raman spectra of PS-beads; (c) Light microscopy of PS-beads (FTIR-ATR-objective); (d) FTIR-spectra of PS-beads (top: 2 µm, bottom: 5µm).
